# Improving quality indicator report cards through Bayesian modeling

**DOI:** 10.1186/1471-2288-8-77

**Published:** 2008-11-18

**Authors:** Byron J Gajewski, Jonathan D Mahnken, Nancy Dunton

**Affiliations:** 1 Department of Biostatistics, School of Medicine, University of Kansas Medical Center, Kansas City, KS, USA; 2 School of Nursing, University of Kansas Medical Center, Kansas City, KS, USA

## Abstract

**Background:**

The National Database for Nursing Quality Indicators^® ^(NDNQI^®^) was established in 1998 to assist hospitals in monitoring indicators of nursing quality (eg, falls and pressure ulcers). Hospitals participating in NDNQI transmit data from nursing units to an NDNQI data repository. Data are summarized and published in reports that allow participating facilities to compare the results for their units with those from other units across the nation. A disadvantage of this reporting scheme is that the sampling variability is not explicit. For example, suppose a small nursing unit that has 2 out of 10 (rate of 20%) patients with pressure ulcers. Should the nursing unit immediately undertake a quality improvement plan because of the rate difference from the national average (7%)?

**Methods:**

In this paper, we propose approximating 95% credible intervals (CrIs) for unit-level data using statistical models that account for the variability in unit rates for report cards.

**Results:**

Bayesian CrIs communicate the level of uncertainty of estimates more clearly to decision makers than other significance tests.

**Conclusion:**

A benefit of this approach is that nursing units would be better able to distinguish problematic or beneficial trends from fluctuations likely due to chance.

## Background

In 1998 the American Nurses Association (ANA) established the National Database of Nursing Quality Indicators (NDNQI) in order to provide hospitals national comparative data (report cards) that measure *nursing *sensitive indicators of quality of care [1]. The NDNQI's primary mission is to provide hospital nursing units a national system for comparing their quality of care, as measured by nurse staffing and nursing-sensitive patient outcomes, to the quality of care among nursing units of the same type in similar hospitals . The NDNQI has grown over the past ten years from 23 hospitals enrolled in 1999 to the current total of 1,350 hospitals reporting data for over 10,000 nursing units. Site coordinators are trained in standardized data collection techniques and data entry. Data are entered quarterly, via a secure website. In return, hospitals receive quarterly report cards for each of their participating nursing units as well as summary statistics for all NDNQI units of the same type in similar hospital types. These comparisons, called benchmarks, allow unit staff to understand how the quality of care on their units compares to similar units elsewhere. Unless otherwise stated, the term "unit" refers to "nursing unit." Hospital administrators use this information to make decisions about whether to make quality improvement changes, for example, increasing the amount of nurse staffing, implementing new risk assessment procedures, or prevention protocols.

The current process does not provide a measure of uncertainty at the unit level and therefore does not support optimal decision making. Historically, NDNQI provides significance information by noting whether a confidence interval covers the unit's value for an indicator. The original reports bold a nursing unit is if it is significantly above or below the overall mean. Technically it is the mean of the same type of units in similar type of hospitals. This confidence interval reflects the average *across units*; susceptible to many of the units being "significantly" higher or lower than the mean. The use of the confidence interval is also problematic because, as the sample size increases, more and more units will have values significantly different from the mean.

Recently, NDNQI held a user focus group to study how hospital staff uses the NDNQI report cards. Paraphrasing one user, "whenever our unit is significantly above the overall mean, we immediately require the quality nurse to explain this deficiency in writing." Further, NDNQI'S technical assistance staff has reported that hospitals' staff voiced a strong concern and need to react when the bolded indicators when their unit showed a statistically significant problem (personal communication, Susan Klaus, 6/25/08). This feedback indicates a possible overreaction to statistically significant difference from the mean.

There are challenges in generating intervals at the unit level. Specifically, unit data based on a small number of patients would be at risk of extreme period-to-period variation due to the occurrence of a rare event. Such variability would not reflect the overall level of the true quality of care provided on the unit. Further, measuring uncertainty can be very difficult if no events are observed.

The literature on model-based report cards indicates that Bayesian hierarchical models are optimal for addressing interpretation problems resulting from small numbers of observations. Indeed, Bayesian hierarchical models have been treated as the "gold standard" because they provide a sound basis for smoothing random variation and for estimating uncertainty in estimates – both of which could reduce over-reaction and possibly costly errant decision making (e.g. [2,3]). The general accepted practice for fitting Bayesian hierarchical models is via Markov chain Monte Carlo (MCMC) estimation [4].

NDNQI report cards are generated quarterly, incorporating approximately 163 measures. Before actually generating reports, data quality is investigated using statistical methods in order to detect outliers, missing data, and illogical data patterns. Nursing units with potential errors in their data are flagged and NDNQI staff calls the hospitals to correct errors. The data are processed using over 2,000 lines of SAS code, which takes 3–4 hours to run. Reports must be issued within 30–45 days of the close of data entry for a quarter.

The literature is filled with arguments advocating for Bayesian hierarchical models. Some recent examples for report cards follow. Reference [5] advocates using hierarchical models for facility profiling in the presence of small sample sizes because one can borrow information to improve estimates. Reference [3] suggests that hierarchical models are very useful in league tables while stressing the importance of adjustment.

Our primary goal was to develop a procedure approximating the fully Bayesian hierarchical method that is easy to implement and is transparent to the hospital report users. A fully Bayesian approach via MCMC is not feasible with NDNQI's deadline for report delivery given the iterative and monitoring requirements of MCMC and the fact that there are 163 indicators and over 10,000 units.

We propose a method for approximating the fully Bayesian approach using modeling frequently referred to as the empirical Bayes approach [6]. We illustrate its utility on NDNQI data with three different indicators: fall rates (e.g. [7]), pressure ulcer rates (e.g. [8]), and Registered Nurse (RN) job enjoyment (e.g. [9]). We discuss these indicators because they reflect diversity in sampling distributions (Poisson, binomial, and normal, respectively) as well as diversity in the method of data collection within the NDNQI. For fall rates, one does not have control over the sample size, but we will see that units could collect more information on pressure ulcers. We will illustrate the Bayesian approach for use in practice by presenting "example report cards." As part of these report cards, we will calculate a "quality index" which is the probability a *unit's *indicator is below the mean of all similar units.

## Methods

### 2.1 Data source

For comparison purposes, the benchmarks used in report cards are stratified by unit type and bed size (or some other hospital characteristic). The unit types include: critical care, step down, medical, surgical, combined medical-surgical, and rehabilitation. The hospitals are stratified into five bed size categories or three teaching status categories. These variables provide the comparison groups for each of the nursing units; for example, all combined medical-surgical units from a small teaching hospital are compared to one another. We do not include the covariates in a large hierarchical model here but create separate models for each subgroup. As previously mentioned, of the 163 measures, we focused on the following three NDNQI indicators: fall rates, pressure ulcer (PrU) rates, and registered nurse job enjoyment (JE). We use data collected in a recent quarter for the combined medical-surgical units. We do not disclose the bed size here (for this paper's example) because specific benchmarks are proprietary data. While we illustrate the methodology on this specific set of indicators, the method can be replicated to other indicators that follow different distributions.

The fall rates are the number of falls per thousand patient days. PrU rates are the number of patients in a 24 hour period that have at least one pressure ulcer as a proportion all patients assessed. Job enjoyment data are from a survey of registered nurses (RN). This indicator is the average of seven questions on a six-point Likert scale, ranging from (1) strongly disagree to (6) strongly agree. Example questions include: nurses are satisfied with jobs and find real enjoyment in their job. We will further define these indicators in Section 2.2. The summary statistics for the three indicators are listed in Table 1. This includes the overall average ($\bar{y}$) variance (*s*^2^) and the number of units (*N*).

**Table 1:** Summary statistics (across medical-surgical units) for each indicator.

Indicator	$\bar{y}$	*s*^2^	*N*
Fall Rates	4.31	4.61	163
Pressure Ulcer (PrU) Rates	0.0553	0.0038	171
Job Enjoyment (JE)	3.49	0.4528	97

We note that many report card systems advocate risk adjustment. Advocates of risk adjustment believe that it allows for fair comparisons across units that may have different patient populations. Alternatives to risk adjustment include defining *a priori *an acceptable indicator rate. NDNQI does not perform risk adjustment. Rather, we stratify by unit type and bed size. In fact, risk adjustment is controversial. Reference [10] provide an interesting history and benefits of risk adjustment as well as recommendations for future work. Reference [11] has produced case-mix adjusted indicators using empirical Bayes methods. Reference [12] advocates for risk adjustment with empirical Bayes hierarchical models for nursing homes. On the other hand, [13] and [14] argue for alternatives to risk adjustment.

### 2.2 Approach

In this section we define the general model for each of the indicators and present fully, approximate, and non-informative Bayesian approaches. We discuss model adequacy and model comparison of the gold standard (fully Bayesian approach) with the approximate approach. We make two points: (1) the primary goal is to provide an interval representing variation within the unit rather than across units; and (2) one can only do this (in general) by borrowing information across units.

#### 2.2.1. General model

Let *y*_*j *_be an indicator for the *j*th unit and let *θ*_*j *_denote the parameter that determines the sampling distribution, or *y*_*j*_|*θ*_*j*_~*f*(*y*_*j*_|*θ*_*j*_). The Bayesian hierarchical model (BHM) assumes that *θ*_*j *_is random with a distribution *Π*(*θ*_*j*_|*θ*_0_), where *θ*_0 _is a vector of hyper-parameters that need to be estimated. The posterior distribution is defined by applying Bayes theorem. Using our notation, the posterior distribution is *θ*_*j*_|*y*_*j*_~*g*(*θ*_*j*_|*y*_*j*_) = *f*(*y*_*j*_|*θ*_*j*_)*Π*(*θ*_*j*_|*θ*_0_)/*m*(*y*_*j*_) where *m*(*y*_*j*_) = ∫*f*(*y*_*j*_|*θ*_*j*_)*Π*(*θ*_*j*_|θ_0_)*dθ*_*j*_. The posterior predictive distribution of the unit (which will be used for goodness of fit), is the sampling distribution integrated across the posterior distribution, specifically *y*^*p*^_*j*_|*y*_*j*_~∫*f*(*y*^*p*^_*j*_|*θ*_*j*_)*g*(*θ*_*j*_|*y*_*j*_)*dθ*_*j*_.

Next we discuss this BHM in the context of our three example indicators: fall rates, pressure ulcers, and RN job enjoyment. For each indicator, we discuss the specific sampling distribution, prior distributions of the parameters, and their posterior distributions. Much of the detail that we discuss here can be found in [4].

#### Poisson (Fall Rates)

For fall rates, we assume that *z*_*j *_is the number of falls across the quarter of interest and *w*_*j *_represents the number of patient days divided by 1,000. The fall rate indicator is then *y*_*j *_= *z*_*j*_/*w*_*j *_which represents the observed number of falls per 1,000 patient days. We assume that *z*_*j *_follows a Poisson distribution and that *θ*_*j *_is the average fall rate (per 1,000 patient days) for the *j*^th ^unit. Therefore, *z*_*j*_|*θ*_*j*_~Poisson(*θ*_*j*_*w*_*j*_). A conjugate prior for the Poisson distribution is the gamma distribution, so we assume that *θ*_*j*_~Γ(*k*,*θ*), where Γ(.,.) is a gamma distribution with mean *kθ *and variance *kθ*^2^. Supposing, for now, that *k *and *θ *are known, then the posterior distribution of *θ*_*j*_|*z*_*j *_is Γ(*z*_*j*_+*k*,1/{*w*_*j*_+1/*θ*}). Therefore, the posterior mean of *θ*_*j*_|*z*_*j *_is (*z*_*j*_+*k*)/(*w*_*j*_+1/*θ*) = {*θw*_*j*_/(*θw*_*j*_+1)}*y*_*j *_+ {1/(*θw*_*j*_+1)}*kθ*, which is a linear combination of the observed fall rate *y*_*j *_and the prior fall rate *kθ*. The term "prior falls" refers to the number of "equivalent" falls informed by the "prior" distribution. This terminology is used throughout the paper.

#### Binomial (Pressure Ulcer Rates)

For hospital acquired pressure ulcers (PrU), let *z*_*j *_be the number of patients out of *n*_*j *_who have a hospital acquired pressure ulcer that is observed during their the 24 hour data collection period. The indicator is then *y*_*j *_= *z*_*j*_/*n*_*j *_which represents the observed pressure ulcer rate. We assume that *z*_*j *_is a binomial distribution with *n*_*j *_trials and that *θ*_*j *_is the average pressure ulcer rate for unit *j*. Therefore, *z*_*j*_|*θ*_*j*_~Bin(*θ*_*j*_,*n*_*j*_). A conjugate prior for the binomial distribution is *θ*_*j*_~Beta(*α*,*β*), where Beta(.,.) is a beta distribution with mean *α*/(*α+β*) and variance *αβ*/[(*α+β*)^2^(*α+β*+1)]. Again, suppose, for now, that *α *and *β *are known, then the posterior distribution of *θ*_*j*_|*z*_*j *_is Beta(*z*_*j*_*+α*,*n*_*j*_-*z*_*j*_+*β*). Therefore, the posterior mean of *θ*_*j*_|*z*_*j *_is (*z*_*j*_*+α*)/(*n*_*j*_*+α*+*β*) = {*n*_*j*_/(*n*_*j*_+*α*+*β*)}*y*_*j*_+{(*α*+*β*)/(*n*_*j*_*+α*+*β*)}*α*/(*α+β*) which is a linear combination of the observed PrU rate *y*_*j *_and the prior PrU rate *α*/(*α+β*).

#### Normal (RN Job Enjoyment)

For RN job enjoyment (JE), let *y*_*j *_be the observed average score and *s*_*j *_the standard deviation for *n*_*j *_RNs in unit *j*. We assume that *y*_*j *_is normally distributed; reasonable in practice despite the fact that *y*_*j *_is bounded because this average rarely reaches the ends of the boundary. We assume that *θ*_*j *_is the average RN job enjoyment for unit *j*. Therefore, *y*_*j*_|*θ*_*j*_~N(*θ*_*j*_,*σ*^2^/*n*_*j*_), where we assume *σ*^2 ^= {Σ(*n*_*j*_-1)*s*_*j *_^2^}/{Σ(*n*_*j*_-1)}. The assumption of homogenous *σ*^2 ^could be relaxed. We assume that *θ*_*j*_~N(*θ,σ*^2^_θ_). The posterior distribution is *θ*_*j*_|*z*_*j*_~N(*θ*_j _*,*V*_j _*) where *θ*_j_* = {*n*_*j*_/*σ*^2^}/{*n*_*j*_/*σ*^2^+1/*σ*^2^_θ_}*y*_*j*_+{1/*σ*^2^_θ_}/{*n*_*j*_/*σ*^2^+1/*σ*^2^_θ_}*θ*, again a linear combination of the observed and prior means, and *V*_j_* = 1/{*n*_*j*_/*σ*^2^+1/*σ*_θ_}.

#### Measures of Uncertainty

Assuming the prior parameters are known, the uncertainty can be summarized by the posterior distribution of *θ*_*j *_using 2.5% and 97.5%-tile as a 95% credible interval (CrI) [4]. In reality, these are unknown posterior distributions and so we would estimate them using a fully Bayesian computation such as MCMC every quarter [4].

#### 2.2.2. Fully Bayesian approach

Bayesian profiling has been in the literature for over ten years (e.g. [15-18]). Reference [19] studies different Bayesian decision rules for profiling hospitals and the methodology was further justified via optimal probability cuts for these decisions in [20].

Agency for Healthcare Research and Quality (AHRQ) [21] recently studied the practicality and the consequences of fitting hierarchical models for performance indicators. One of the conclusions of the workgroup was that it was clear that these models were "gold standard," but there were practical limitations in these iterative methods including computing time and the monitoring of convergence. Therefore, the following approximate (empirical) Bayesian approach was assessed. This approximate approach was derived from summary statistics from the most recent NDNQI report card data.

#### 2.2.3. Approximate Bayesian approach

Empirical Bayes methods for profiling were developed by [22]. Empirical Bayes in the health literature has a long history ([23-26]). Following the standard empirical Bayes approach, we do the following to estimate hyper-parameters of all models. For each of the outcomes, define $\bar{y}$ = Σ*y*_*j*_/*N *and let *s*^2 ^= Σ(*y*_*j*_-$\bar{y}$)^2^/(*N*-1). The statistics $\bar{y}$ and *s*^2 ^are both summarized in the current report cards. Next, for the purposes of approximating hyper-parameters, for each of the three outcomes above, temporarily set *θ*_*j*_. = *y*_*j*_. Then use the method of moments (MOM) (e.g. [27]) to find estimates of the parameters of the prior distributions.

Because the MOM estimates of the prior distribution are specified by summary statistics, the MOM estimates for fall rates are 1/*θ *= $\bar{y}$/*s*^2 ^and *k *= $\bar{y}$^2^/*s*^2 ^telling us that the higher the mean or the lower the variance is, the more "equivalent prior" number of patient days. If the variation (*s*^2^) is small, then we can assume that other units are providing more information.

The MOM for the prior PrU has a similar property, the solution is *α *= $\bar{y}${$\bar{y}$(1-$\bar{y}$)/*s*^2^-1} and *β *= (1-$\bar{y}$){$\bar{y}$(1-$\bar{y}$)/*s*^2^-1}. Considering that *α *is the prior number of pressure ulcers and *α*+*β *the prior sample size, again we can see that as $\bar{y}$ increases the prior PrU rate goes up and as *s*^2 ^decreases the prior sample size increases.

The interpretation for job enjoyment is straightforward with usual normal theory as *θ *= $\bar{y}$ and *σ*^2^_θ _= *s*^2^. The lower *s*^2 ^is the more informative the prior. Further, recall that the posterior variance of the unit is *V*_j_* = 1/{*n*_*j*_/*σ*^2^+1/*σ*^2^_θ_} = *σ*^2^/{*n*_*j*_+*σ*^2^/*σ*^2^_θ_} arguing that *σ*^2^/*σ*^2^_θ _is the prior sample size.

The approximate Bayesian approach produces results similar to, but slightly more conservative than, the fully Bayesian approach. This is because the variance of the hyper-parameters under the fully Bayesian approach essentially estimate the variance of the smoothed parameters through the shrinkage estimates. The prior parameters under the approximate Bayesian approach do not use any sort of shrinkage, therefore resulting in larger variances for the prior distribution compared to the fully Bayesian approach.

#### 2.2.4. Non-informative Bayesian approach

A third approach uses what is sometimes called non-informative or a flat prior distribution. Essentially, one assumes that there is no other information outside of the summary statistics observed for the particular unit types under question and that there are no prior patients for pressure ulcers, no prior patient days, and that the variance of the prior distribution for JE is infinity. This results in CrIs close to the traditional confidence intervals. A drawback of the approach is that it is very difficult to calculate intervals when information is observed on the edge of the sample space (e.g. 0 falls, 0 PrU, or *n*_*j *_PrU).

#### 2.2.5. Model adequacy and relative fit

To test whether the models were correctly specified, we calculate a chi-square goodness of fit measure for each of the indicators using the fully Bayesian approach (Gelman et al, 2000). Specifically, we define χ^2 ^= Σ(*y*_*j*_-*θ*_*j*_)^2^/*Var*(*θ*_*j*_) and χ^2^_*p *_= Σ(*y*^*p*^_*j*_-*θ*_*j*_)^2^/*Var*(*θ*_*j*_) for the posterior predictive data. The discrepancy between model parameters and the observed data is χ^2 ^and for the posterior predictive data is χ^2^_*p*_. The goodness of fit Bayesian p-value is thus Pr(χ^2 ^< χ^2^_*p*_) and values that are between 0.01 and 0.99 are deemed to reflect a reasonable fit. There is no need to incorporate the degrees of freedom in the calculation because the probability is calculated using the posterior distribution from MCMC.

To test how well the approximate Bayes approach emulated the fully Bayesian approach we utilize the Deviance Information Criterion (DIC) ([28]), which is an ad-hoc alternative to Bayes' factor that involves the likelihood and a penalty term. The lower the DIC the better the relative fit. We look at the relative fit for all indicators using DIC for: (i) the fully Bayesian approach; (ii) the approximate Bayesian approach; and (iii) a non-informative approach, which emulated a traditional confidence interval (CI) approach.

We look at the number of times we would decide a unit's indicator is "significantly" below (or above) the overall mean across all units. For the purposes of this paper we will decide a unit is significantly below the national mean if Pr(*θ*_*j *_<$\bar{y}$|*y*_*j*_,*θ*_0_) > .95 (above if Pr(*θ*_*j *_> $\bar{y}$|*y*_*j*_,*θ*_0_) > .95). We defined a quality index for the *j*^th ^unit to be *Q*_*j *_= Pr(*θ*_*j *_> $\bar{y}$|*y*_*j*_,*θ*_0_) for JE and reverse the inequality to *Q*_*j *_= Pr(*θ*_*j *_<$\bar{y}$|*y*_*j*_,*θ*_0_) for PrU and fall rates. We calculated *Q*_*j *_for the approximate Bayesian approach and a non-informative approach.

#### 2.2.6. Sensitivity of Sample Size

The limitations of the approximate relative to the fully Bayesian approach was explored by varying the number of nursing units in the analysis. Using randomly selected sample sizes of 5, 10, 25, 50, 75, and 95, we compared the approximate approach to the fully Bayesian approach by taking 10,000 draws from the posterior prediction of a future nursing unit using the approximate parameters from the method of moments and comparing against 10,000 draws from the fully Bayesian approach. This was repeated for fall rates, PrU rates, and JE. The goal was to see at what point we would be "forced" to use a fully Bayesian approach rather than an approximation.

## Results

The fully Bayesian approach was implemented using MCMC with the program WinBUGS. The hyper parameters had vague priors: fall rates *k*~Γ(0.01,100), 1/*θ*~Γ(0.01,100); PrU rates *α*~Γ(0.01,100), *β*~Γ(0.01,100); and JE *θ*_*j*_~N(*θ,σ*^2^_θ_)*θ*~N(0,31.6^2^), 1/*σ*^2^_θ_~Γ(0.001,1000). Alternatively, weakly informative priors (not considered here) might be useful. The prior mean, for example, could be centered at the units' sample mean from the prior quarter. The prior variance could be based on expectations about how far a unit is likely to differ from the average in an extreme case. Using the fully Bayesian approaches, the model across all three indicators were adequate as measured by Bayesian p-values. The model adequacy is summarized by p-values that indicate whether the model is an accurate reflection of the data. If the p-value is high, then we are inclined to believe that the model is adequate. If the p-value is low then we will reject the adequacy of the model and would need to fit an alternative approach. These p-values were p = 0.5189 for fall rates, p = 0.4686 for PrUs, and p = 0.5184 for JE, which indicated that the Poisson, binomial, and normal distributions were adequate models for the sampling distribution. We report model adequacy for the fully Bayesian approach only since the other approaches are its approximations.

Table 2 shows the DIC across the fully, approximate, and non-informative Bayesian models. The DIC for the fully and the approximate Bayesian approach were within 10 for each of the indicators. We observed a much greater improvement in the DIC of these approximate Bayes models relative to the non-informative models. Table 2 offers evidence that an approximate Bayesian approach is adequate relative to the gold standard and a considerable improvement over a model that does not borrow any information.

**Table 2:** Summary of relative fit across models for each indicator.

Indicator	Sampling Distribution	DIC, fully	DIC, approximate	DIC, non-informative
Fall Rates	Poisson	909.2	907.3	965.9
PrU Rates	Binomial	498.1	490.8	572.3
JE	Normal	61.2	61.0	87.4

**Figure 1:**
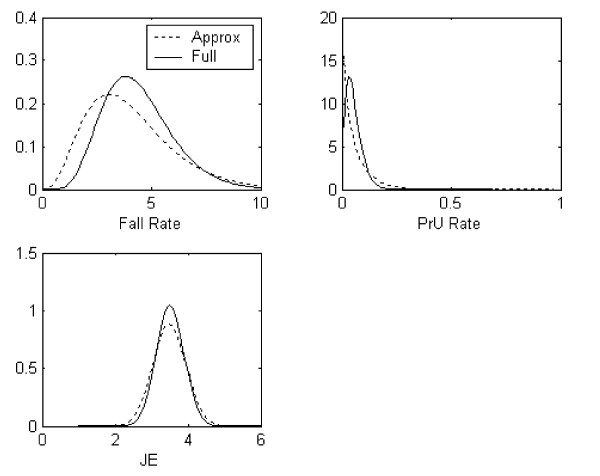
Prior distributions for three indicators using the Full and Approximate Bayesian Models.

To further describe the differences between the fully and approximate approaches we plotted the prior distributions for each indicator (Figure 1). Consistent with the DIC analysis, we see that these distributions approximately overlapped and that the tails from the approximate Bayesian models were heavier than their fully Bayesian comparisons. Let us focus on the MOM estimates and how these relate to prior information. The MOM estimates for fall rates are 1/*θ *= $\bar{y}$/*s*^2 ^= 4.31/4.61 = 0.93 and *k *= $\bar{y}$^2^/*s*^2 ^= 4.31^2^/4.61 = 4.02 telling us the information from other units provides around one-thousand patient days (around 11 patients per 24 hour days) and just over 4 falls. For the prior PrU the solution is *α *= $\bar{y}${$\bar{y}$(1-$\bar{y}$)/*s*^2^-1} = 0.70 and *β *= (1-$\bar{y}$){$\bar{y}$(1-$\bar{y}$)/*s*^2^-1} = 12.04, corresponding to 0.70 as the number of prior pressure ulcers and almost 13 as the prior number of patients. The interpretation for job enjoyment is straightforward with usual normal theory as *θ *= 3.49 and *σ*^2^_θ _= 0.45. The pooled within unit variance is *σ*^2 ^= 0.71; thus the prior sample size estimate is *σ*^2^/*σ*^2^_θ _= 0.71/0.45 = 1.58 (a prior of 1.5 RNs). We can demonstrate the relative amount of information borrowed (on average) by taking the ratio of the prior patient days and the average patient days, ratio of prior sample size and average sample size, and the ratio of the prior RNs and the average RNs. This corresponds to 0.93/2.31 = 0.40; 12.74/24.1 = 0.53; and 1.58/16.6 = 0.10, respectively. These results indicate that the information across units for fall rates and pressure ulcers informs individual units more than they do for JE.

**Figure 2:**
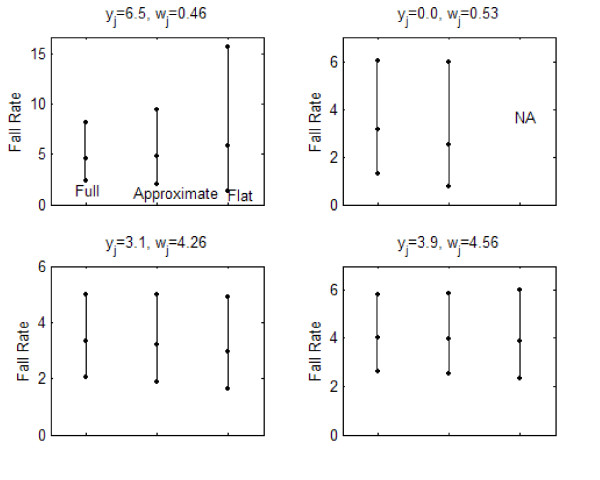
Posterior distribution for four units' fall rates.

**Figure 3:**
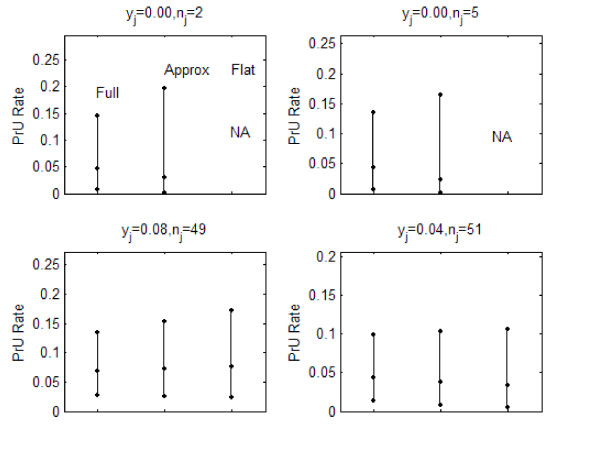
Posterior distribution for four units' PrU rates.

**Figure 4:**
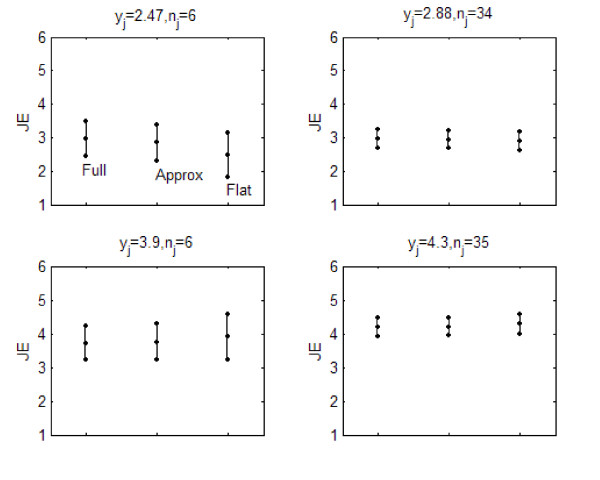
Posterior distribution for four units' for JE.

Figures 2, 3, and 4 describe the posterior distribution for several units for all three indicators using the posterior 2.5, 50.0, and 97.5-%tiles. For fall rates, the posterior for the full and approximate credible intervals were similar. The non-informative (flat) had wider intervals than those of the other methods except for the unit that reported zero falls because its interval could not be calculated. PrU rates demonstrated a similar phenomenon. For JE all of the intervals were similar, with the non-informative (flat) being slightly wider than the others.

On a personal computer with 3.20 GHz and 2.00 GB RAM, the fully Bayesian approach took 27 seconds to sample 11,000 MCMC iterations. Assuming similar number of units across 163 indicators, the method would take 73 minutes; a small time savings. The real savings occurs because the approximate Bayes approach does not require monitoring of convergence of the MCMC and is thus much easier to automate the approximate Bayes approach for report card generation. Additionally, the approximate approach is easier to explain to NDNQI users. A switch to the full Bayes approach would be necessary in the event that the approximate approach inadequately reflects the full approach, which would require continued assessment of this relationship for future indicators.

**Figure 5:**
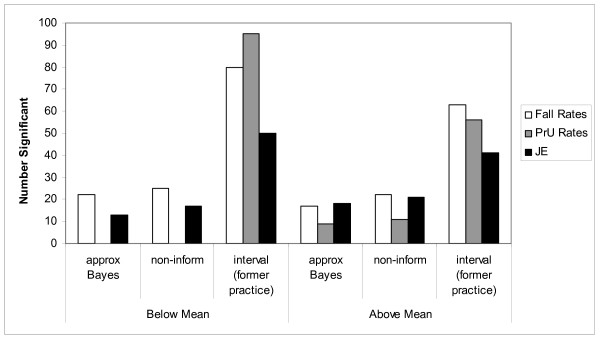
Comparison of methods for assessing significant units.

Overall (Figure 5), using the approximate Bayesian method there were 22 units that had fall rates significantly below the overall mean and 17 units that had fall rates significantly above the overall mean. Conversely, using a method that is non-informative there were 25 and 22 significantly different units respectively. These results indicate that 8 units could make the wrong decision – over-reacting by saying a unit is below or above the national mean. Further, there were three more units under the non-informative approach that we would be unable to calculate confidence intervals for because there were 0 falls. For PrU rates the counts were 0 and 9 for the approximate 0 and 11 for non-informative methods (61 unable to calculate). The results were mixed for JE. For JE the counts were 13 and 18 for the approximate and 17 and 21 for the non-informative methods (0 unable to calculate).

These results are in stark contrast to what one gets using an interval approach across units (indicator is significant if above or below this interval). The 95% confidence interval for the overall mean for falls, PrUs, and JE was 3.97–4.63; 0.046–0.065; and 3.56–3.63 respectively corresponding to 80 below and 63 above for falls; 95 below and 56 above for PrUs; and 50 below and 41 above for JE.

### 3.1 Example Report Cards

The following displays represent how different reports would look for two different units for fall rates and PrU rates.

#### Display for Unit X (Fall Rates)

In the last quarter, a unit X had 8 falls and 2,348 patient-days. This resulted in an observed fall rate of 3.41 falls per thousand patient days. A 95% credible interval for the unit was 2.15–5.96. The average across all units of this type was 4.30. The quality index for fall rates was thus: 0.75. The quality index is the probability that a unit's fall rate is below the overall average, with a higher score being better. We consider units with a quality index above 0.95 to be significant. The fall rate on this unit was not significantly below the average fall rate.

#### Display for Unit Y (Fall Rates)

In the last quarter, a unit Y had 1 fall and had 1,481 patient-days. This resulted in an observed fall rate of 0.68 falls per thousand patient days. A 95% credible interval for the unit was 0.68–4.25. The average across all units of this type was 4.30. The quality index for fall rates was thus: 0.98. The unit's fall rate was significantly below the average.

#### Display for Unit X (PrU Rates)

In the last quarter, unit X had 3 patients with PrUs out of 24 patients in the census. This resulted in an observed PrU rate of 0.13. A 95% credible interval for unit X was 0.03–0.22. The average across all units of this type was 0.06. The quality index for fall rates was thus: 0.19. The unit was not significantly below the average PrU rate.

#### Display for Unit Y (PrU Rates)

In the last quarter, unit Y had 0 patients with PrUs out of 17 patients in the census. This resulted in an observed PrU rate of 0.00. A 95% credible interval for the unit was 0.00–0.10. The average across all units of this type was 0.06. The quality index for fall rates was thus: 0.89. The unit was not significantly below the average PrU rate.

### 3.2 Fully versus Approximate Bayes for Various Sample Sizes

**Figure 6:**
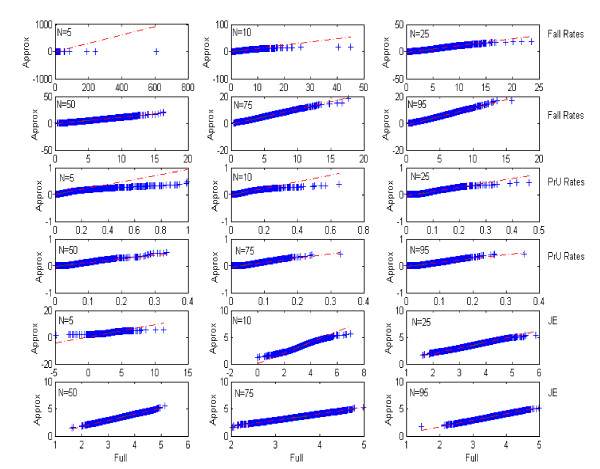
Q-Q plots of 10,000 simulations comparing "full" Bayesian approach to the "approx" Bayesian approach for sample sizes varying from 5 to 95.

Figure 6 shows the q-q plots for the approximate versus fully Bayesian approaches across all indicators. In all cases, there was strong evidence that the approximate approach was valid at *N *= 25 nursing units and above. Below that point (*N *= 5 & 10) the method of moments tended to underestimate the tails. This inequality diminished after 25; which suggested that using the approximate method only when there are more than 25 nursing units in the subset used for report card generation is advisable.

## Discussion

The intent of this study was to explore a practical approximation for implementing a Bayesian approach for nursing outcome report cards. This method supplies report card users with more information than was given in the past reports; specifically, the probability of being below the overall mean and the 95% CrI. This represents a methodological and informational improvement. The examples demonstrated the utility of this approach for determining exemplary performance. As an alternative to the quality index, a deficiency index could similarly be derived. Use of a deficiency index could prove beneficial in reducing the chances of over-reacting through the incorporation of prior information into the index. Additionally, Bayesian hierarchical models handle multiplicity automatically. Multiplicity could be defined as lowering the Type I error rate – the probability of identifying units that are "significantly" lower than the overall mean. Note that Austin and Brunner (2007) recommend different probability levels, but for the purposes of this paper we use a 0.95 probability level.

There are several consequences and extensions to our study that are worth noting.

### Point 1: sample size calculations

The example data suggests that the fully Bayesian approach can guide us in generating policies for gathering information. Some indicators provide more information than others. The PrU data, collected in one 24-hour period, has relatively lower sample sizes than fall rates, which are collected over all days in a quarter. Our method suggests policy changes such as requesting units to conduct more prevalence studies each quarter rather than just one; but our experience suggests that this will not happen until most facilities in the U.S. have electronic medical records from which performance measures can be extracted. This policy could shorten CrIs and supply units with more precise information. Currently, there are some hospitals in NDNQI that conduct as many as three prevalence studies per quarter and they are implicitly rewarded with more stable indicators that have relatively narrow CrIs.

### Point 2: temporal analysis

NDNQI reports provide data for each unit across eight quarters. We can extend our approach to make smooth estimates across time. (A drawback of smoothing is that when there is a meaningful change it is masked.) Let *θ*_*jk *_be the parameter for the *j*th unit where *k = *1,2,3...,8 quarters of data. Calculate a posterior distribution of *θ*_*j*1 _using the approximate Bayesian approaches but then use it as *an individual *prior for *θ*_*j*2 _and then continue on such that after the first step, each previous posterior is a prior for the next time point. This type of model is an approximation to a Kalman filter or a state space model (e.g. [29]). Notice that according to this method, prior information is accumulating, thus in order to down weight the past, after *k *= 2, we weigh the previous information by 1/2 of its prior information. This seems a sensible alternative to a more complicated and computationally expensive model.

### Point 3: overall summary of quality

Suppose we want to combine the quality indicators from falls, pressure ulcers, and job enjoyment into one value we call overall quality summary. Suppose, *conditional on unit*, the quality indicators tend to be approximately uncorrelated – or they provide a unique perspective of quality. It seems reasonable that the posterior distributions of the indicator parameters are independent. We can combine information about the quality index. For example, suppose we want to calculate the probability that the unit is above the overall mean on *both *fall rates and PrU rates. For unit X, the overall quality summary is: 0.75^*^0.19 = 0.14 and unit Y it is 0.98^*^0.89 = 0.87. Indicating that unit Y has evidence of better overall quality than X. However, we may need to incorporate a dependent structure as we may expect various outcome indicators to be correlated because of the quality of nursing care on the unit.

## Conclusion

This analysis has demonstrated that approximate Bayesian CrIs will communicate the level of uncertainty of estimates more clearly to decision makers than other significance tests because the large sample sizes in NDNQI reports can lead to very small standard errors. In this context, significant differences from the mean may not be clinically important and the effect of random change in the prevalence of adverse events exaggerated by traditional approaches.

How will users interpret the proposed method? Will they understand CrIs? Will they use the new information? The answers to these questions may not be straightforward. We intend to address them with a small pilot study. The best indicator of success will be when units initiate quality improvements based on accurate interpretation of report card information – rather than on chance fluctuation – after being presented with summaries from an approximate Bayesian approach compared to units who use report cards summarized from traditional approaches. The expectation is that this will occur because units will be less likely to react to chance and more likely to act upon more complete information about their quality of care. Our proposed method has a good statistical foundation and is practical to implement. We think this will be transparent to our users and can be implemented in a spread sheet program like Excel. We show all the 2003 Excel functions needed to implement the approximate Bayesian approach in the appendix.

## Appendix: Excel functions for approximate Bayesian approach

1. = average()

2. = stdev()

3. = GAMMAINV(,,,)

4. = BETAINV(,,,)

5. = NORMINV(,,,)

6. = GAMMADIST(,,,)

7. = BETADIST(,,,)

8. = NORMDIST(,,,)

## Competing interests

Partial funding for all authors is with a contract from the American Nurses Association (ANA) who fund the National Database of Nursing Quality Indicators (PI: Dunton).

## Authors' contributions

BG conceived the study, performed statistical computing/modeling and drafted the manuscript. ND contributed the substantive interpretations. JM contributed statistical modeling ideas. All authors read and approved the final manuscript and contributed to the ideas of the study as well as to editing and re-writing.

## Pre-publication history

The pre-publication history for this paper can be accessed here:


